# *Dystroglycan 1:* A new candidate gene for patellar luxation in Chihuahua dogs

**DOI:** 10.14202/vetworld.2018.1277-1284

**Published:** 2018-09-17

**Authors:** Pattarawadee Srinarang, Korakot Nganvongpanit, Waranee Pradit, Kittisak Buddhachat, Puntita Siengdee, Kumpanart Soontornvipart, Siriwadee Chomdej

**Affiliations:** 1 Department of Biology, Faculty of Science, Chiang Mai University, Chiang Mai 50200, Thailand; 2 Department of Veterinary Biosciences and Public Health, Animal Bone and Joint Research Laboratory, Faculty of Veterinary Medicine, Chiang Mai University, Chiang Mai 50100, Thailand; 3 Excellence Center in Osteology Research and Training Center, Chiang Mai University, Chiang Mai 50100, Thailand; 4 Science and Technology Research Institute, Chiang Mai University, Chiang Mai 50200, Thailand; 5 Department of Biology, Faculty of Science, Naresuan University, Phitsanulok 65000, Thailand; 6 Department of Veterinary Surgery, Faculty of Veterinary Science, Chulalongkorn University, Bangkok 10330, Thailand

**Keywords:** DNA marker, *Dystroglycan 1* gene, inter simple sequence repeat, patellar luxation, single-nucleotide polymorphism

## Abstract

**Aim:**

The objective of this study was to uncover new candidate genes related to patellar luxation (PL) in dogs to select for those with low susceptibility for breeding purposes.

**Materials and Methods:**

The inter simple sequence repeat (ISSR) technique was performed to construct DNA fingerprints of 61 Chihuahua dogs with PL and 30 healthy Chihuahua dogs. DNA polymorphisms were detected by comparing the sequences between the affected and unaffected dogs, using the pairwise alignments in MultAlin. Genotyping was performed using allele-specific polymerase chain reaction (AS-PCR). The association analysis of ISSR DNA fingerprints and genotypes or phenotypes was performed using the Chi-square (*χ*^2^) model and generalized linear model (GLM), respectively.

**Results:**

Two single nucleotide polymorphisms (SNPs), namely SNP1UBC811 (g.91175C>G) and SNP2UBC811 (g.92259T>C), were found in the intron of the *Dystroglycan 1* (*DAG1*) gene, which was obtained using the PL-related marker UBC811 primer (p=0.02), and genotyped by AS-PCR. When investigated using the GLM, g.91175C>G had a significant association with PL (p=0.0424), whereas g.92259T>C did not have such an association (p=0.0959).

**Conclusion:**

*DAG1* might be one of the genes related to PL in Chihuahuas and could aid the process of marker-assisted selection in genetic breeding for Chihuahua dogs without PL.

## Introduction

Patellar luxation (PL) is a non-infectious disease related to the bone, tendons, and muscles. It is often found in miniature breed dogs such as Pomeranians, Chihuahuas, and Boston Terriers, as well as in young dogs [1], and likely arises due to mutations in multiple genes. The occurrence of PL is approximately twice as high in females than in males (the ratio of female-to-male is 1.95:1) [2], and in 82% of reported cases, it is an inherited disorder [3]. In dogs with PL, the patella is dislocated from the trochlear groove of the femur, toward either the inside (medial) or outside (lateral), causing pain and inability to move the legs at regular intervals. Previously, a retrospective study of PL prevalence conducted in an orthopedic clinic in Chiang Mai during the period from October 2006 to May 2011 recorded that 128 of 317 dogs (40.3%) were diagnosed with PL, 34.3% of these were small dogs (Poodle), and most of them were female (57.8%) [1]. The onset of the disorder can be noted at an early age, and both the canine breed and sex influence the presence of PL, suggesting that it is inherited [4]. Wangdee *et al*. [5] showed that the heritability (h^2^) of PL in Pomeranian dogs was 0.44, indicating that PL is genetically influenced. Although PL in dogs is thought to be a disease that is regulated by multiple genes, very little is known about the pattern of genetic control and inheritance [6]. For these reasons, it is necessary to find the causative or relevant genes related to the disease.

As miniature breeds of dogs have become increasingly popular as pets, breeders have become concerned with improving the health status of these dogs. Understanding the onset of PL and discovering the genes that are relevant to PL will help to genetically improve canine breeds with desirable traits. DNA fingerprint techniques such as random amplified polymorphic DNA (RAPD) and inter simple sequence repeat (ISSR) are common tools for generating DNA fragments for marker-assisted selection (MAS), for example, detection of DNA markers in dogs with PL by high annealing temperature-RAPD [7] and the use of ISSR to identify markers at clusters of disease resistance genes [8]. Furthermore, ISSR is a quick and straightforward method that can simultaneously amplify the DNA fragments between two microsatellite regions from the whole genome without prior knowledge of the genome sequence [9].

In this study, the aim was to identify DNA-based markers associated with the presence of PL in Chihuahua dogs using ISSR. It was hypothesized that the ISSR technique would be able to find the DNA markers associated with the PL phenotype in dogs.

## Materials and Methods

### Ethical approval

The present study was approved by the Ethics Committee of the Faculty of Veterinary Medicine, Chiang Mai University, Thailand, in 2016 (approval letter number: S36/2559).

### PL diagnosis and collection of blood samples

**Figure-1:**
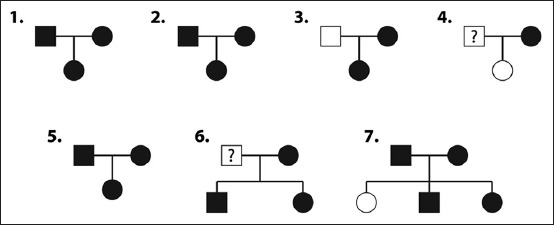
The pedigree of Chihuahua dogs. Squares and circles represent male and female dogs, respectively. Filled symbols indicate dogs with patellar luxation, empty symbols indicate dogs with no patellar luxation, and squares with a question mark denote unknown.

Ninety-one dog blood samples of Chihuahua dogs (61 affected and 30 unaffected) were used in this study. Most samples were from unrelated individuals, but some samples were from known family relationships as shown in the pedigree (Figure-1). The data, including age, sex, and grade of disease, were recorded, and all the blood samples were kept at −20°C, before DNA extraction. The PL grading system according to Wangdee *et al*. [5] was used and is briefly described here. In Grade I PL, the patella can be manually dislocated during full extension of the stifle joint and return to the normal position when released. In Grade II PL, the patella gets dislocated more frequently than in Grade I. The patella gets dislocated easily, especially when the foot and tibia are rotated, and is pushed in a medial or pulled in a lateral direction. Returns to the normal position occur with the opposite procedure. In Grade III PL, the patella is permanently dislocated but can manually be returned to the normal position when the stifle is extended; however, flexion and extension of the stifle result in relaxation of the patella. In Grade IV PL, the patella is permanently dislocated and cannot be manually repositioned.

### Inclusion and exclusion criteria for samples

The dogs belonging to the affected group were Chihuahua dogs with clinical signs of medial PL (all grades). Animals that were pregnant, had a neurological disease, or had undergone musculoskeletal surgery were excluded. Dogs with lameness due to cranial cruciate ligament rupture or meniscal injury and those with nerve injury, lumbosacral instability, infection, immune diseases, or fractures were also excluded from the study. The dogs belonging to the normal group were Chihuahua dogs with more than 1 year of age and without clinical signs of PL.

### DNA extraction

The isolation of the total genomic DNA for analysis was carried out by employing the phenol-chloroform method, with slight modifications [7]. A volume of 1 ml of Tris-acetate EDTA (TAE) buffer was added to a 1.5 ml microcentrifuge tube containing a 200 µl blood sample. The mixture was vortexed and centrifuged at 6000 rpm for 5 min. The supernatant was discarded and 500 µl of lysis buffer with 5 µl of 20 mg/ml proteinase K and 50 µl of 10% sodium dodecyl sulfate was added. The samples were incubated at 65°C overnight until the pellets were digested. A mixture of 500 µl of phenol-chloroform was added and mixed by inverting before centrifuging at 12,000 rpm for 10 min. The supernatant was transferred into a fresh 1.5 ml tube with 500 µl of chloroform and mixed by inverting before centrifuging at 12,000 rpm for 10 min. The supernatant was transferred into another fresh 1.5 ml tube. Double volume of absolute ethanol and a volume of 3 M sodium acetate were added. The mixture was incubated at −20°C overnight and centrifuged at 13,000 rpm for 15 min. The supernatant was poured out, and 1 ml of 70% ethanol was added. Centrifugation at 13,000 rpm for 10 min was carried out. The supernatant was poured out, and the pellet was dried in a 65°C incubator for 30 min. The dry pellet was suspended in 20 µl ddH_2_O, and the DNA suspension was stored at −20°C until used. The DNA was qualified and quantified using BioDrop DuO (BioDrop, England).

### ISSR analysis

The DNA of dogs was pooled for primer screening and optimization for ISSR analysis. The PL group was the DNA pooled from PL Grades III and IV of up to 10 individual samples (10 ng/μl of each sample) and the N group was the DNA pooled from normal DNA of up to 10 individual samples (10 ng/μl of each sample), regardless of age and sex.

Polymerase chain reaction (PCR) was performed in a total volume of 25 μl containing the following: 1× reaction buffer (160 mM (NH_4_)_2_SO_4_, 500 mM Tris-HCl [pH 9.2 at 22°C], 17.5 mM MgCl_2_, and 0.1% Triton™ X-100 [Vivantis Technologies, Malaysia]), 0.25 mM deoxynucleotide triphosphate (dNTP) (Vivantis Technologies, Malaysia), 0.4 μM primers (UBC primer set 9 Biotechnology Laboratory, The University of British Columbia, Canada) (Table-1), 1 U *Taq* DNA polymerase (Vivantis Technologies, Malaysia), and 10 ng of genomic DNA; distilled water was added to this to make a final volume of 25 μl. PCR reagent was done using a Major Cycler, CYCLER-25 Thermal Cycler (Major Science, USA), with the following cycling profile: 1 cycle at 95°C for 5 min; 38 cycles at 94°C for 45 s, 55°C for 45 s, and 72°C for 45 s; and a final cycle at 72°C for 5 min. After the PCR was completed, the amplified samples were separated by 1.5% agarose gel with 3X GelRed (Biotium, USA) using 1× TAE buffer under constant 120 V for 45 min and then visualized under a UV illuminator.

**Table-1:** List of primers and their sequences used in ISSR analysis, number of fragments, and size ranges.

Primer	Sequence (5’ 3’)	Total number of amplified fragments	Total number of polymorphic fragments	Fragment size range (bp)
UBC801	ATA TAT ATA TAT ATA TT	0	0	-
UBC802	ATA TAT ATA TAT ATA Tg	0	0	-
UBC803	ATA TAT ATA TAT ATA TC	0	0	-
UBC805	TAT ATA TAT ATA TAT AC	0	0	-
UBC807	AgA gAg AgA gAg AgA gT	15	0	400-2500
UBC808	AgA gAg AgA gAg AgA gC	16	0	500-2000
UBC809	AgA gAg AgA gAg AgA gg	0	0	-
UBC811	gAg AgA gAg AgA gAg AC	15	4	400-1500
UBC817	CAC ACA CAC ACA CAC AA	18	2	400-1500
UBC818	CAC ACA CAC ACA CAC Ag	15	0	400-1500
UBC822	TCT CTC TCT CTC TCT CA	0	0	-
UBC823	TCT CTC TCT CTC TCT CC	0	0	-
UBC824	TCT CTC TCT CTC TCT Cg	0	0	-
UBC825	ACA CAC ACA CAC ACA CT	15	3	400-1750
UBC826	ACA CAC ACA CAC ACA CC	14	2	400-1500
UBC827	ACA CAC ACA CAC ACA Cg	9	3	300-1500
UBC835	AgA gAg AgA gAg AgA gYC	12	0	300-1500
UBC844	CTC TCT CTC TCT CTC TRC	13	0	300-1500
UBC845	TCT CTC TCT CTC TCT CRg	0	0	-
UBC847	CAC ACA CAC ACA CAC ARC	10	0	400-1500
UBC848	CAC ACA CAC ACA CAC ARg	0	0	-
Total		152	14	300-2500

ISSR=Inter simple sequence repeat

The appearance of each polymorphic fragment, DNA markers present in one group (affected or unaffected by PL) but absent in others, was scored as 0 or 1 for its occurrence or absence, respectively, regardless of its intensity. The association between PL and polymorphic DNA fragments was evaluated using the Chi-square (*χ*^2^) model [10].

### Cloning and sequencing of DNA fragments

Candidate fragments obtained from ISSR analysis were excised, using a clean scalpel, and purified using NucleoSpin^®^ Gel and PCR clean-up protocol (Macherey-Nagel, Düren, Germany). The candidate fragments were ligated with the RBC TA Cloning Vector Kit using T4 DNA Ligase enzyme (RBC Bioscience, Taiwan) following the manufacturer’s instructions. The fragment-ligated vectors were transformed into HIT-DH5α competent cells (RBC Bioscience, Taiwan). The mixture was incubated on ice for 10 min and poured immediately onto previously prepared Luria-Bertani (LB) broth agar. Positive colonies were selected by the blue/white colony selection technique and were confirmed for the inserted DNA fragments by colony PCR using M13 forward and reverse primers. The colony that gave the correct DNA fragment length was inoculated in LB broth by vigorous shaking (200 rpm) at 37°C overnight. The plasmid was extracted using the PureYield™ Plasmid Miniprep System (Promega, Fitchburg WI, USA) and was sequenced by an automated sequencer (1^st^ BASE, Malaysia).

### Single-nucleotide polymorphism (SNP) identification

The sequences of the two fragments (PL and N) were blasted with the GenBank dog (*Canis lupus familiaris*) genome database using the BLAST algorithm. DNA polymorphisms were detected by comparing the sequences between the PL-affected and unaffected dogs, using the pairwise alignments in MultAlin (http://multalin.toulouse.inra.fr/multalin/).

### Genotyping by allele-specific PCR (AS-PCR)

To study the association between SNPs and PL, AS-PCR was carried out. Primer pairs for SNP genotyping were designed using Primer Premier 3 and Primer BLAST as shown in Table-2. AS-PCR reactions using specific primer pairs for each allele were carried out in a 25 μl volume containing the following: 1× reaction buffer (16 mM (NH_4_)_2_SO_4_, 50 mM Tris-HCl [pH 9.2 at 22°C], 1.75 mM MgCl_2_, and 0.01% Triton™ X-100) [Vivantis Technologies, Malaysia]), 0.25 mM dNTP (Vivantis Technologies, Malaysia), 0.4 μM of forward and reverse primers (Table-2), 1 U *Taq* DNA polymerase (Vivantis Technologies, Malaysia), 10 ng genomic DNA, and distilled water. PCR was performed using a Major Cycler, CYCLER-25 Thermal Cycler (Major Science, USA), with the following cycling profile: 1 cycle at 95°C for 5 min; 38 cycles at 94°C for 45 s, 55°C for 45 s, and 72°C for 45 s; and a final cycle at 72°C for 5 min. After the PCR was completed, the amplified samples were separated by 1.5% agarose gel electrophoresis.

**Table-2:** Allele-specific primers, sequences, sizes, and annealing temperatures of each SNP.

Primer	Sequence (5’ 3’)	PCR product size (bp)	Annealing temperature (°C)
SNP1D	Fw: GAGAGAGAGAGAGA**G**ACGAT	467	60
	Rw: GATTCCTTTCTGAGTTTGGTAGG		
SNP1ND	Fw: GAGAGAGAGAGAGA**C**ACGAT	234	60
	Rw: TCTGAACAAGCAAGAAAGTGC		
SNP2D	Fw: GAGCTACCCAGGCAACCCAC	447	60
	Rw: TCCATGCAGGG**G**GCCTGAT		
SNP2ND	Fw: GCATGCACTTTCTTGCTTGTTCAG	361	61
	Rw: TCCATGCAGGG**A**GCCTGAT		

Underline and boldface on nucleotide indicates SNP position. SNP=Single.nucleotide polymorphism, PCR=Polymerase chain reaction

### Analysis of association

The association between genotypes and other phenotypes including PL condition, age, sex, and grades of PL was evaluated by the generalized linear model (GLM) [11] using the R studio version 3.4.1 software. Furthermore, the odds ratio was used to measure the strength of the association between each SNP and the PL condition [12].

## Results

### Phenotype description

Of the 30 male, 57 female, and 4 sex-unknown Chihuahua dogs with a range of ages (*X̄*=34.65 months, standard deviation [SD]=22.49, *X̄* and SD of the PL group were 34.87 and 22.65, respectively, *X̄* and SD of the normal group were 34.65 and 22.53, respectively), sex, and grades, 67.03% had PL and the others were normal. Overall, 80% of the male dogs and 57.9% of the female dogs were affected with PL. Most of the dogs with PL (36%) had PL Grade 3 (Table-3), and the phenotype of some PL samples in this study showed that the pedigree of Chihuahua dogs with PL was unable to indicate the mode of inheritance of the disease (Figure-1).

**Table-3:** Severity of patellar luxation in Chihuahua dogs.

Groups	Male n=30 (%)	Female n=57 (%)	Unknown n=4 (%)	All n=91 (%)
Normal	6 (20)	24 (42.1)	0 (0)	30 (32.97)
PL positive	24 (80)	33 (57.9)	4 (100)	61 (67.03)
Grade 1	2 (6.67)	7 (12.29)	1 (25)	10 (11)
Grade 2	6 (20)	8 (14.04)	0 (0)	14 (15.38)
Grade 3	14 (46.67)	16 (28.07)	3 (75)	33 (36.26)
Grade 4	2 (6.67)	2 (3.5)	0 (0)	4 (4.4)

The male and female percentages were calculated using the total of the respective sex. PL=Patellar luxation

### ISSR analysis

**Figure-2:**
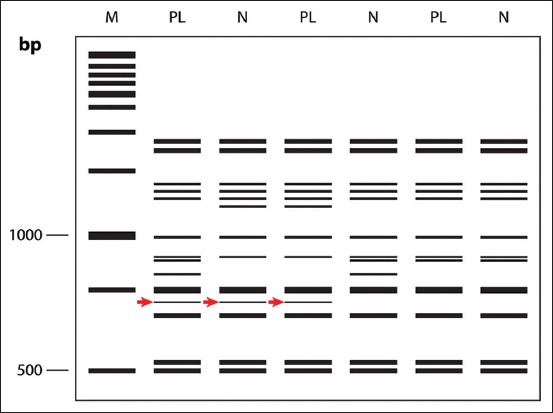
DNA fingerprints from ISSR using UBC811 primer in patellar luxation (PL) dogs and normal (N) dogs, using 1.5% agarose gel stained with GelRed (Biotium, USA) and photographed by a UV transilluminator under UV light (M-1kb plus DNA Ladder [Fermentas, USA]) The red arrows indicate a polymorphic fragment that was found.

A total of 21 ISSR primers were used for the initial screening to find the DNA fragments of the samples of dogs with and without PL. A total of 146 DNA fragments were produced by these primers. These included UBC807, UBC808, UBC811, UBC817, UBC818, UBC825, UBC826, UBC827, UBC835, UBC844, and UBC845. Fourteen of 152 fragments were polymorphic bands yielded by UBC811, UBC817, UBC825, UBC826, and UBC827, which varied in size from 300 bp to 2500 bp, as shown in Table-1. Among the polymorphic bands, the 607 bp fragment produced from the UBC811 primer showed a significant association with PL status (*χ*^2^, p=0.02) (Figure-2).

### SNP identification and genotyping

**Figure-3:**
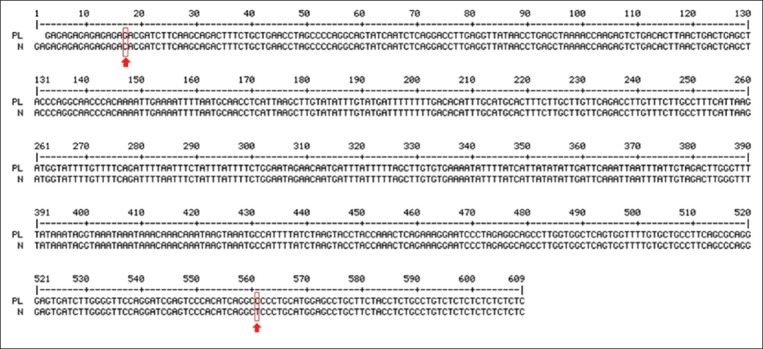
Alignment of the sample with patellar luxation (PL) and the normal (N) sample. The numbers refer to the nucleotide position, and the red arrows indicate the single-nucleotide polymorphisms found in these fragments.

The DNA fragments of 607 bp obtained from the PL and non-PL samples were sequenced. The nucleotides were 83% identical to the intron of the canine *Dystroglycan 1* (*DAG1*) gene. Two SNPs were observed in the fragment (Figure-3): (i) There was an SNP at the primer binding site with a transversion substitution, C>G, namely SNP1UBC811 (g.91175C>G), and (ii) there was another one located at the end of that fragment, with a transition substitution, T>C, namely SNP2UBC811 (g.92259T>C).

**Figure-4:**
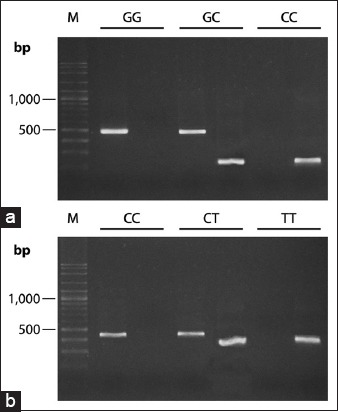
Genotyping of single-nucleotide polymorphism (SNP) by allele-specific polymerase chain reaction of two loci as in SNP C>G of the SNP1UBC811 (a) and T>C of the SNP2UBC811 (b), using 1.5% agarose gel stained with GelRed (Biotium, USA) and photographed by a UV transilluminator under UV illuminators.

AS-PCR was used for genotyping the two SNPs in the Chihuahua population. Three genotypic patterns: GG, GC, and CC of SNP1UBC811 (g.91175C>G) and CC, CT, and TT of SNP2UBC811 (g.92259T>C) were noted (Figure-4). The most frequent genotyping of both SNP1UBC811 and SNP2UBC811 in the population was heterozygous, as shown in Table-4.

**Table-4:** Genotypic and allelic frequencies of SNP1UBC811 and SNP2UBC811.

SNP	Genotype frequency			Allele frequency	
SNP1UBC811	GG	GC	CC	f (G)	f (C)
	0.15	0.79	0.06	0.54	0.46
SNP2UBC811	CC	CT	TT	f (C)	f (T)
	0.04	0.58	0.38	0.33	0.67

SNP=Single-nucleotide polymorphism

### Analysis of association

The association analysis by the GLM of the health status (PL and non-PL) and various phenotypes showed that age (p=0.669) and sex (p=0.155) had no relationship to PL. A significant association was found for SNP1UBC811 (p=0.0424), but no association was found for SNP2UBC811 (p=0.0959. The GG and GC genotypes of SNP1UBC811 led to an increased risk of developing PL. As for SNP2UBC811, the CT and TT genotypes showed a significant association with PL (Table-5). In addition, the odds ratio was used to analyze the association of each genotype to the appearance of PL. It was found that the GC genotype of SNP1UBC811 and the CT genotype of SNP2UBC811 were strongly associated with the presence of PL (6.42-fold) compared to the GG genotype of SNP1UBC811 (95% confidence interval [CI]=1.477-27.87) and 4.213-fold compared to the TT genotype of SNP2UBC811 (95% CI=1.44-12.32) (Table-6). At the same time, the genotype CC of both the SNPs influenced the presence of PL, similar to the GG genotype of SNP1UBC811 or the TT genotype of SNP2UBC811.

**Table-5:** Analysis of the association of patellar luxation in Chihuahua dogs with genotypes of SNP1UBC811 and SNP2UBC811, using a generalized linear model.

Phenotype	SNP1UBC811			SNP2UBC811		
	
	GG	GC	CC	CC	CT	TT
Status	0.014724*	0.012*	0.61894	0.246	0.00479**	0.016556*
Sex	0.9931	0.993748	0.996	0.871	0.0983	0.0808
Age	0.986	0.518	0.661	0.661	0.765	0.90266

* Indicates significant difference at p<0.05.

** indicates a significant difference at p<0.01

**Table-6:** Odds ratio of genotype and patellar luxation condition of SNP1UBC811 and SNP2UBC811

SNPs	PL	N	OR	p-value
			
SNP1UBC811	(48)	(24)	95% CI	
GC	44 (91.67%)	16 (66.67%)	6.4167	0.0131**
			1.477-27.87	
CC	1 (2.08%)	1 (4.17%)	2.3333	0.5901
			0.1068-50.99
GG	3 (6.25%)	7 (29.17%)	Reference	

**SNP2UBC811**	**PL**	**N**	**OR**	**p-value**
		
	**(48)**	**(24)**	**95% CI**	

CC	1 (2.08%)	2 (8.33%)	0.54167	0.6342
			0.0433-6.769	
CT	35 (72.92%)	9 (37.5%)	4.213	0.0086**
			1.44-12.32
TT	12 (25%)	13 (54.17%)	Reference	

* Indicates significant difference at p<0.05.

** indicates a significant difference at p<0.01. SNP=Single-nucleotide polymorphism, PL=Patellar luxation

## Discussion

The findings of this study reveal two SNPs in the intron of the *DAG1* gene, SNP1UBC811 (g.91175C>G) and SNP2UBC811 (g.92259T>C), that show a strong association with PL in Chihuahua dogs.

Based on the ISSR fingerprints from Chihuahua populations with and without PL, a DNA marker related to the presence of PL was observed, and its nucleotide sequence was 83% identical to canine *DAG1*. The *DAG1* gene encodes dystroglycan-alpha (DAG-α), or 156DAG, which, in dogs, is located on chromosome number 20q15.1-q15.2, whereas in humans and mice, it is on chromosome 3p21 and chromosome 9F, respectively. DAG-α is a central component of the dystrophin-glycoprotein complex (DGC) (or called dystrophin-associated protein complex) and plays a role in the connection between the intracellular actin cytoskeleton (through dystrophin interactions) and extracellular matrix (ECM) components such as laminin (basal lamina) in the skeleton muscle [13,14]. The dramatic reduction of 156DAG-α in Duchenne muscular dystrophy (DMD) leads to dysfunction of DGC, rendering muscle fibers more susceptible to necrosis [13]. In addition, a previous study reported that differentiated skeletal muscle from mice with the disruption in the *DAG-α* gene exhibited abnormal muscle regeneration and a degenerative muscular disease called muscular dystrophy [15].

In the current study, two SNPs were found in the DNA marker: g.91175C>G and g.92259T>C. AS-PCR was then allowed to genotype each Chihuahua dog for the two SNPs. The results of the GLM and the odds ratio analysis suggest that the GC and CT genotypes of g.91175C>G and g.92259T>C, respectively, are significantly associated with PL occurrence in Chihuahua compared to other genotypes. Although the SNPs observed in this study are located on the intron of the *DAG1* gene, the two SNPs might be linked to mutation points within other essential elements of the *DAG1* gene such as exons or regulatory elements. Unfortunately, the association analysis using the haplotype data (the types of the two combined SNPs) was not performed because there was no sequence data; this will have to be done in further studies.

Several recent studies have identified candidate genes related to PL in different dog breeds. A genome-wide association study (GWAS) of Pomeranian dogs [5] showed that the *SC5D* gene on chromosome 5 and the *BMPR1B* gene on chromosome 32 were significantly associated with medial PL. In Dutch Flat-Coated Retrievers, *TNR* on chromosome 7 coding for Tenascin R is a candidate gene for PL due to the identification of a synonymous variant [4] by GWAS. These findings indicate that the susceptibility to PL of different breeds might be different.

**Figure-5:**
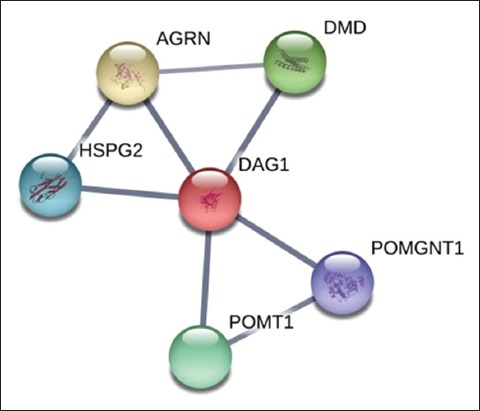
The interacting proteins for the DAG1 gene using the STRING version 10.5 (https://string-db.org): dystroglycan (DAG1), heparan sulfate proteoglycan 2 (HSPG2), agrin (AGRN), dystrophin (DMD), protein O-mannosyltransferase 1 (POMT1), and protein O-linked mannose beta1, 2-N-acetylglucosaminyltransferase (POMGnT1).

Analysis of the interacting proteins of the *DAG1* gene and other genes using the STRING version 10.5 (https://string-db.org) found that dystroglycan (DAG1) interacts with heparan sulfate proteoglycan 2 (HSPG2), agrin (AGRN), dystrophin (DMD), protein O-mannosyltransferase 1 (POMT1), and protein O-linked mannose beta1,2-N-acetylglucosaminyltransferase (POMGnT1) (Figure-5).

HSPG2, AGRN, DMD, and DAG1 are components of the DGC, and DAG-α has been shown to bind to AGRN and HSPG2 in the ECM at the neuromuscular junction [16]. In skeletal muscle fibers, the DGC plays an essential structural role by linking the cytoskeletal protein dystrophin to laminin in the ECM. Mutations that affect any of the proteins involved in this complex lead to myofiber degeneration and are associated with muscular dystrophies and congenital myopathies. Since the loss of dystrophin in DMD leads to an almost complete loss of DAG complexes at the myofiber membrane, it is generally assumed that the vast majority of DAG-α complexes within skeletal muscle fibers interact with dystrophin [17].

In contrast, a mutation in *POMT1* and *POMT2* in humans and mammals causes a group of congenital muscular dystrophies; the most severe of these autosomal recessive condition is the Walker–Warburg syndrome, which occurs due to reduced O-glycosylation of DAG-α (hypoglycosylation) in post-translational modification [18]. In addition, abnormally glycosylated DAG also causes mutation in *POMGnT1*, which leads to an autosomal recessive disorder, called muscle–eye–brain disease [19].

Our GLM and odds ratio analysis demonstrated that the presence of PL was equally frequent in male and female. This is consistent with a previous study that found that PL is equally common in male and female Pomeranian dogs with a relative risk of 1.11 (95% CI 0.98-1.25) [5]. In contrast, a study by Soontornvipart *et al*. [2] showed that the presence of PL in female Pomeranian dogs is approximately twice as high than in male Pomeranian dogs. We also found no association between age and PL occurrence, although PL prevalence has often been reported to occur in young dogs [1]. This could stem from the difficulty in observing PL symptoms in dogs in the early stages because the symptoms are more clearly observed in the severe stages of the disease.

Here, the ISSR technique was used for discovering a gene marker linked to the appearance of PL in dogs. This is an effective molecular tool for DNA fingerprinting as it offers high reproducibility and stability and does not require prior DNA information; for these reasons, it is widely used for identifying DNA-based markers for disease resistance, temperature-sensitive male sterility, and agronomic traits in plants [9].

## Conclusion

In sum, a DNA-based marker derived from ISSR was obtained for identifying a candidate gene involved in PL. We identified two SNPs, namely g.91175C>G and g.92259T>C, situated on the intron of the *DAG1* gene on chromosome 20 and revealed that the GC and TT genotypes of the two SNPs, respectively, are significantly associated with the appearance of PL in Chihuahua dogs. Furthermore, we found that there was no association between sex or age and PL in Chihuahua dogs. Future studies should scan for the mutation point on the *DAG1* gene of Chihuahua dogs to ascertain its role in PL and could aid the process of MAS in genetic breeding for Chihuahua dogs without PL.

## Authors’ Contributions

SC and KN supervised and designed the experiment. KS provided the samples. PSr carried out experimental work and wrote the manuscript. WP, KB, and PSi participated in the organization of laboratory work and data analysis. All authors have read and approved the manuscript.
